# Verification of the Perception of the Local Community concerning Air Quality Using ADMS-Roads Modeling

**DOI:** 10.3390/ijerph191710908

**Published:** 2022-09-01

**Authors:** Kinga Szopińska, Agnieszka Cienciała, Agnieszka Bieda, Janusz Kwiecień, Łukasz Kulesza, Piotr Parzych

**Affiliations:** 1 Faculty of Civil and Environmental Engineering and Architecture, Bydgoszcz University of Science and Technology, 85-796 Bydgoszcz, Poland; 2 Faculty of Environmental, Geomatic and Energy Engineering, Kielce University of Technology, 25-314 Kielce, Poland; 3 Faculty of Geo-Data Science, Geodesy and Environmental Engineering, AGH University of Science and Technology in Kraków, 30-059 Krakow, Poland

**Keywords:** air quality, surveys, GIS, pollution modeling, Smart City

## Abstract

Road transport is one among the sources of air pollution in a city, which results in lowering the comfort of life and increases the occurrence of respiratory diseases. The level of pollutants emitted in the city is variable, and it depends on the type and nature of the source and the manner of land development. For this reason, the purpose of the article is an attempt at a spatial (inner) diversification of a city in terms of air quality, using a study of perception and semantic differentials (SD). The research, which covered the period from June to November 2021, was performed in Kielce—the Polish Smart City—among local experts, people well acquainted with the city and knowledgeable about air quality and the impact of pollution on human health. The results allowed the demarcation of areas with the best and the worst parameters in terms of air quality within the city. Verification of the survey was carried out using the ADMS-Roads (Atmospheric Dispersion Modeling System) software for modeling pollution levels and GIS software, using data on road traffic. The verification allowed checking whether the respondents participating in the research accurately evaluated the city space. The modeling proved that within the two selected areas, the pollution level is similar, and it does not exceed the permitted values. This might indicate that in society there is still low awareness of air quality, particularly in terms of knowing the sources of pollutants and their impact on human health, and perception of areas with the best and the worst air quality was the result of an analysis of the manner of land development and its morphology.

## 1. Introduction

According to the WHO, 90 percent of urban residents breathe air that does not meet the standards for health [1]. As indicated by Nahorski and Holnicki [2], air pollution is the source of health risk for a major part of the population due to a huge increase in the population of cities, where concentrations of harmful compounds in the air are usually much higher. Urbanization leads to rapid changes in the landscape, causing both positive results as well as considerable threats and problems. This phenomenon significantly affects the environment [3], quality of life [4], and the structure and functioning of the city [5]. Road transport is the main linear source of atmospheric pollution in cities [6,7], next to other point sources, which include pollution from industrial sources (chimneys) as well as from municipal and household sources, related to individual heating as well as the accumulation and disposal of waste [8]. A dynamic increase in the number of vehicles driving on the roads, which is accompanied by a slow development of the transport network [9], causes a noticeable drop in the average speed of vehicle traffic. Road networks are incapable of efficient handling of the generated traffic, which causes an increase in the air pollution level and a higher frequency of respiratory diseases among the inhabitants. Road transport is a direct source of the atmospheric emission of gaseous pollutants and particulates generated due to the combustion of liquid fuels as well as the wearing of brakes and tires. It is also a source of secondary particulates generated in the process of condensation of gaseous substances (sulfates, nitrates, and hydrocarbons) emitted in exhaust fumes, as well as secondary lifting of particles deposited on the road surface [10]. The magnitude and type of emissions of pollutants present in exhaust fumes are related to numerous factors, and they depend on the type and power of the engine, the extent to which it is worn, the applied solutions reducing the emission (a catalytic converter, a particulate filter), the operating conditions (driving speed, the phase of motion), meteorological conditions, the type and condition of the road surface and the composition of fuel [11]. As is currently believed [1,12,13], road transport is the most significant source of the following pollutants in the air: NO_2_, CO, C_6_H_6_, and PM, especially with respect to the sub-micron matter PM1.0 originating from the engines, and the coarse matter PM2.5–10 originating from the wearing of brake linings, tires, and road surfaces. According to the provisions of the Cleaner Air for Europe (CAFE) directive [14], it is necessary to implement observations and tools which would allow for distinguishing between factors with a significant impact on the current air quality and the possibility of its improvement. In European countries, air quality standards implemented via permitted levels or target levels are in effect for 12 air pollutants: SO_2_, NO_2_, CO, PM10, PM2.5, Pb, C_6_H_6_, and O_3_, as well as for polycyclic aromatic hydrocarbons (PAHs) and heavy metals (As, Cd, Ni), for which it is necessary to monitor both the concentrations in the air, as well as deposition on the ground. There are various attitudes aiming at the reduction of air pollution. Some of them concern integrating transport and land-use policies. Cities such as London and Oxford have created Low Emission Zones (LEZs), where the most polluting vehicles are prohibited. In some LEZs, the most polluting vehicles have to pay more if they enter the area. In other locations, alternatives such as carpool lanes, park and ride stations or subsidized public transport for students are implemented, as well as actions discouraging the use of personal vehicles such as implementing fuel (car) or pollution taxes and parking fees. Among others, Chile has expanded its electric bus fleet and committed to cutting black carbon emissions. Moreover, a lot of cities, including Dublin and London, have established car-free days, encouraging the residents to run, walk and cycle [15,16].

Remedying actions are very important, but each one of them must be preceded by proper monitoring, which is necessary in effective policy regarding air quality. In all EU countries, air quality assessment is performed in accordance with the procedure specified in the CAFE directive [14], and it is related to a zone which is within the administrative borders of a city with the number of inhabitants exceeding 100 thousand. The level of harmful substances is determined, and the following classification is introduced for the entire zone. Class A—air quality in the city is good; the level of substances does not exceed the permitted level or the target level for the substances. Class C—air quality in the city is bad; the level of at least one substance exceeds the permitted level increased by a margin of tolerance, or the target level. Cities assigned to class C require remedying actions, aimed at improving air quality by implementing an air protection program in the zone. The classification applies to the whole city, with no internal spatial differentiation.

Currently, pollution maps [17], which present the average level of pollution in a given time interval, are the main diagnostic tool used to analyze air quality in a city [18]. Next to pollution maps, the forecasting uses new methods, based, for example, on neural networks [19,20] or the land-use regression (LUR) technique for air pollution modeling, in order to obtain the spatial distribution of air pollutants for epidemiological studies [21]. As emphasized by Targino et al. [22], Zhan et al. [23] and Pattinson et al. [24], the evaluation of exposure and health effects with the use of site-monitoring NO_2_ data is uncertain, especially for regions distant from the measurements. Furthermore, unpredictable pollution events may occur, such as fires, construction sites and changes in the weather conditions, that may have negative impact on the reliability of these maps. Hasenfratz et al. [25], referring to, among others, Jerrett et al. [26] and Zheng et al. [27,28], indicate that, in order to predict intraurban pollution concentrations, various kinds of models can be developed, for example, statistical interpolation, LUR, data mining techniques, proximity-based assessment, line dispersion models, and novel machine learning. Such prediction models, assessed as sensible, have been applied on an EU-wide scale for NO_2_, PM10, and O_3_ [29]. Li et al. [30] developed a Gaussian process regression model (also known as Kriging) on the basis of land-use characteristics, in order to estimate urban ultrafine particulate (UFP) levels. Jensen, Berkowicz, et al. [31] emphasize that in Denmark, decision-support GIS tools for urban air quality and human exposures management are based on, among other things, cadastral digital maps, the Danish operational street pollution model (OSPM), and Danish national administrative databases concerning the population. As the authors have found, ambient air pollution levels are estimated at high temporal and spatial resolutions, enabling the mapping of traffic emissions, air quality levels and human exposures at residence addresses, at workplace addresses and in the streets. Unfortunately, pollution maps still commonly used by city authorities are prepared based on measurement data collected from sensors, the number of which is insufficient for a complete examination of the pollution level in the city [13]. Moreover, the use of data recorded by a network of monitoring stations results in the uncertainty of modeling [32], and it requires the integration of information supplied by deterministic models based on the meteorology of emission and the chemical and physical characteristics of the air atmosphere [33,34,35]. Meanwhile, there are many studies in urban environments, as well as rural ones that do not have any measurements provided, so that the creation of complete maps with high resolution is a tough challenge. In fact, traffic-related pollutants in urban environments can vary substantially within a few meters [22,24]. Uncertainty is inevitably encountered when evaluating exposure and health effects while using site-monitoring NO_2_ data, especially for regions far from the measurement sites [22,23]. Taking into account only the data from measurement stations prevents detailed estimation of the air pollution level in a city (zone) [11]. During the preparation of pollution maps, the variability of sources or the variability of morphology and spatial development of a city are omitted, as noticed by a Chinese research team [36]. Moreover, assessments to date have not considered inhabitants’ opinions about air quality, which should be included in the assessment procedure, especially in areas where bad quality is indicated. After all, the outcome of the assessment is the most important for the inhabitants as the “end receivers”, since it influences their quality of life. Analyses of the subject literature [11,37,38,39] indicate that air quality assessment should be performed using modern measuring and computational techniques, taking into account the variability of sources and the variability of land morphology and development in the city. Referring to the air quality assessments for the individual pollutants as zones (understood as entire cities) provides an incorrect result in the assessment as confirmed by the studies performed in Poland by the research team from Bydgoszcz [11]. Likewise, conclusions about air quality based on the data acquired from sensors mounted at measurement stations or remote sensing data, and the analyses of these data related to a zone (the whole city), are insufficient and subject to errors [11]. In multifunctional urban centers, spatial variability is so high that studies of air quality should be more detailed. The monitoring of air quality for various land uses, including the areas along any transportation routes, is essential, and enables the application of a suitable approach to, among others, planning the locations of residential or green areas in cities, and the appropriate layout of districts. Evaluation of the influence of the lay of the land on the conditions of air could outline a future position on the issue of spatial planning and supporting activities in the field of actions intended to nullify the effects of pollution on the quality of life.

Due to the above, it is the purpose of the article to attempt spatial (inner) differentiation of a city in terms of air quality. The following were performed as part of the defined research objective:

1. Description of an original method for inner differentiation of a city in terms of air quality, using data on road traffic and taking into account the opinions of the inhabitants.
2. Verification of the described method in one of the major cities of Poland by:
  - Demarcation of those areas of the city which have been indicated as the best and the worst by the people well acquainted with the analyzed space in the air quality perception survey.
  - Modeling the level of air pollution with NO_2_ emitted by the road transport by means of advanced GIS technologies and air pollution modeling software.
  - Checking whether the respondents participating in the study of air quality perception properly assessed the city space, and whether they are aware of the factors which affect it negatively.

The research was performed in Kielce, which is the Polish Smart City. Initially, the available source materials related to air quality were studied using document analysis and criticism, including the data originating from the Copernicus Sentinel-5P satellite, dedicated to atmospheric monitoring and downloaded by means of the Google Earth Engine platform. This process was successfully used to assess the level of NO_2_ in Iran [40]. The performed analysis indicated the lack of data about the internal variance of the level of air pollutants. For this reason, a survey was used to achieve the research objective, meaning an attempt at internal differentiation of air quality. Semantic differentials (SD) comparing the individual research areas were formulated based on the produced results. They were used to pinpoint the areas with the worst and the best rating (according to the respondents), meaning those considered to be areas with bad and good air quality, respectively. Verification of the results of the survey was performed with the use of advanced GIS technologies and the ADMS-Roads software for modeling the air pollution level. Verification and detailed assessment of air quality were carried out for the level of NO_2_ generated from the sources of road transport. The choice of the type of pollution was the result of research performed by an international research team [41], who claimed that pollution with NO_2_ is one of the main causes of premature mortality of the inhabitants of Kielce (out of 858 cities studied with respect to premature mortality, Kielce is 44th). The studies also used the ArcGIS Geostatistical Analyst tool, GeoMedia Prof. software, QGIS software and Statistica software.

## 2. Materials and Methods

### 2.1. Study Area

**Figure 1:**
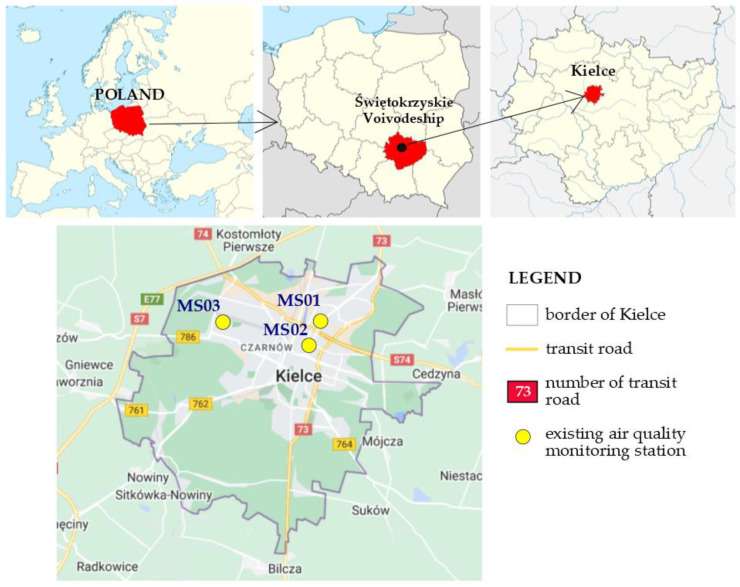
Location of Kielce against the background of maps of Europe and Poland, along with the location of existing air quality monitoring stations.

Kielce is a city of about 200,000 inhabitants, situated in the south-east of Poland (Figure 1). Kielce is the capital of Świętokrzyskie Voivodeship, situated in the Holy Cross Mountains, and is characterized by variable land topography—the difference in relative elevation is 181 m (the highest hill—Telegraf: 408 mamsl, the lowest point—Bobrza river near Słowik: 227.0 mamsl). The city constitutes an important traffic node of Świętokrzyskie Voivodeship, and the main direction of vehicle traffic is along the south-north axis, leading from Krakow (an important academic, cultural, touristic, administrative, and industrial center) to Warsaw (the capital of the country). The main types of economic activity in the city include extraction and processing of mineral resources (iron, copper, and lead ore, marble, and sandstone), the metal and machine industry as well as the building, electromechanical or processing industry, which constitute potential emission sources of air pollutants. Because of the conditions of the terrain and the transport service, the industry is concentrated in the peripheral parts of the city, within the Kielce Technology Park, which reduces the emission pressure of industrial sources. For this reason, the primary emission of air pollutants in the city originates from point sources (municipal and household), associated with individual heating, as well as from road transport.

In the City of Kielce, air quality tests and assessments are carried out as part of the state environmental monitoring conducted by the Provincial Environmental Protection Inspectorate. There are three functional air quality monitoring stations of the urban type in Kielce. Two of them are located in the city center, with another one at the outskirts of the city (Figure 1). The measurement of nitrogen dioxide is carried out at two of them (MS01—the station at no. 108 Warszawska St. and MS02—the station at no. 3 Targowa St.). The former was commissioned in 2018, the latter in early 2021 (Table 1). The measurements recorded by the two stations constitute a basis for the annual assessment of air quality in terms of NO_2_ in the city [42].

The choice of Kielce city as the study area is not accidental, since for several years it has been successfully implementing the ideas of a Smart City—a sustainable intelligent city—and it even received several awards for the achievements in that regard (including at the Smart City World Congress in Barcelona, in 2012). In 2017, the Smart City 2030+ Framework Strategy was created, the purpose of which is activation for joint inspirations and actions executed for increasing the quality of life in the city. When analyzing the subject of the broadly understood quality of life in a Smart City, the issue of air quality can be distinguished as one of the important factors influencing the healthy and proper functioning of the inhabitants [43]. For this reason, it seems strongly justified to determine the air quality in this city in a detailed manner. This will allow the local authorities to implement the principles of sustainable development even more effectively, and to take more efficient actions related to air protection.

### 2.2. Research Methodology and Data

The methodology involved the following steps:

1. Analysis of source materials in the field of air quality in Kielce city, including data from the Copernicus Sentinel-5P satellite using the Google Earth Engine platform, data from the Provincial Inspectorate for Environmental Protection (Polish institution dealing with air quality in the city of Kielce), and data from the air quality monitoring system in Kielce, established in 2020 as part of the “Kielce without smog” [44] and “Smog Kielce” [45] projects.
2. Performing a survey among experts in Kielce (the people closely familiar with Kielce and with knowledge about air quality and the impact of pollution on human health) in order to identify research areas which can be considered to be those with bad and good air quality. The results of the surveys were processed using the quantitative method for studying the assessment of air quality perception—semantic differential (SD).
3. In selected research areas (with the best and the worst air quality)—the identification of objects affecting the quality of air, meaning pollution sources, land morphology, the type of building design, etc., and the indication of those among the areas selected in the survey which are to be considered to be those with the worst and the best quality in the city.
4. Checking the appropriateness of the selection of two areas considered to have bad and good air quality, using advanced GIS technologies and computer software for modeling the level of air pollutants. With consideration for the research results [41] and the predominance of linear sources, verification was carried out for the NO_2_ pollution generated by the road transport:
  - Determining the average daily traffic and the annual average daily traffic (AADT) for each section of the road (link).
  - Calculating nitrogen dioxide emissions with the use of environmental software (ADMS-Roads model) for every road section
  - Creating ADMS results in the ArcMap software: producing contour plots, displaying existing point sources (monitoring stations)
  - Using Microsoft Excel to create a time-series graph of NO_2_ concentration values for the monitoring stations

#### 2.2.1. Analysis of Source Materials in the Field of Air Quality Regarding NO_2_ in Kielce City

The air quality in Kielce can be assessed using the data originating from several sources. The assessment used the data acquired by the Copernicus Sentinel-5P satellite, launched on 13 October 2017. This is the first satellite of the Copernicus constellation dedicated to atmospheric monitoring. Because of the Tropomi instrument (an advanced multispectral imaging spectrometer), it provides detailed and precise data about the atmosphere. The data are shared free of charge with a frequency of 0.01 degrees, for general use [46]. They were downloaded by means of the Google Earth Engine platform. The access to the data is provided using scripts in the Java programming language.

In Kielce city, the assessment of air quality was also performed under the National Environmental Monitoring, carried out by the Provincial Inspectorate for Environmental Protection in Kielce. The report on air quality assessment in Świętokrzyskie Voivodeship [47] is prepared every year based on the results of measurements performed at air monitoring stations located in the entire voivodeship (a total of 14 monitoring stations, including nine that carry out measurements using automatic or automatic and manual methods, and five that only use the manual methods). In Kielce city, there are three monitoring stations (Kusocińskiego St.—a station of the suburban type, Targowa St. and Warszawska St.—stations of the urban type). In the report, air quality assessment is supplemented by mathematical modeling of transport and substance transformations in the air. The preparation of the report in question is an obligation resulting from the provisions of the EU law, implemented in the national law, and one of the purposes of air quality assessment is to determine the probable reasons behind the existence of excessive concentrations of pollutants in specific regions (within a range possible to obtain based on the possessed information). In 2020, the assessment of air quality in Kielce city included about a dozen standardized air pollutants: SO_2_, NO_2_ as well as NO_X_, CO, O_3_, C_6_H_6_, the PM10 particulate matter, the PM2.5 particulate matter, and the presence of metals in the PM10 particulate. The calculations of pollutant concentrations near the ground surface used the GEM-AQ air quality model, which was developed based on the GEM numerical model of weather forecasts (Global Environmental Multiscale), developed and operated by the Canadian Meteorological Centre. Under the MAQNet project, the meteorological model has been extended by introducing a comprehensive troposphere chemistry module. An integral part of the GEM-AQ model is the aerosol module, which allows for simulations of the physicochemical changes in the atmospheric aerosol and its interactions with the chemical compounds of the gaseous phase. Calculations using the GEM-AQ model and the analyses performed for the needs of supporting the annual air quality assessment in Poland were carried out in two stages in a global grid with a variable resolution, the resolution over Poland with a wide margin being 2.5 km (0.025 degrees), while the resolution used for 30 urban agglomerations and cities > 100 thousand inhabitants was 0.5 km (0.005 degrees). The so-called objective estimation was one of the complementary methods was used for the needs of annual air quality assessment in Świętokrzyskie Voivodeship. The estimation methods were used for the needs of determining the spatial distribution of the concentration of selected pollutants over the area of the zone in 2020. The report also distinguished pollutants generated by various sources, including point and transport-related.

Additionally, resolution no. XXXIX/758/2021 of 21 January 2021 on the adoption of the “Programme for Limiting Low-Altitude Emission in Kielce city” [48] is currently in force in the area of Kielce, which is a consequence of the resolution of the Regional Assembly of Świętokrzyskie Voivodeship of 29 June 2020 No. XXII/291/20 on the establishment of the ‘Programme of air protection for Świętokrzyskie Voivodeship with a plan of short-term actions’.

Moreover, information about the air quality in Kielce can be acquired from the “Idea Kielce” web portal for the communication and cooperation of local authorities with the local community [44], allowing for the participation of inhabitants in integrated management of the city. As part of the portal, there is an available system for monitoring air quality, which was created under the “Kielce without smog” project of the 2020 Kielce Participatory Budget. The system consists of 21 devices for measuring air quality, monitoring the concentration levels of the PM1, PM2.5, and PM10 particulate material, mounted in various parts of the city on the facades of public utility buildings. The data originating from the measurements are processed and shared by a widget presenting the results on a web page. The system has an informational and educational nature, and its purpose is to increase awareness of air quality in the city among the inhabitants. As part of a citizens’ initiative in Kielce, another web portal called “Smog Kielce” was created, with information about air quality in the city [45]. Under the project, another network of sensors was mounted in Kielce, measuring the concentration of the PM2.5 and PM10 particulates which are harmful to human health.

#### 2.2.2. Studying the Assessment of Air Quality Perception—Semantic Differential (SD)

An analysis of the abovementioned sources indicates the inability to internally differentiate the city space in terms of air quality. The achievement of this spatial differentiation was attempted by studying the perception of air quality. The survey had a targeted nature, and the respondents were local experts, closely familiar with Kielce and with good knowledge about air quality and the impact of pollution on human health. The respondents originated from the academic environment, the private sector, public services (city hall employees) and the non-profit sector. The criteria for selecting the respondents included their knowledge and experience of the subject in question. Studies with a similar nature (using local experts) were successfully implemented by an international research team performing research in South Africa [49] and India [50].

To select areas with bad and good air quality, a quantitative method called the semantic differential (SD) was used, which examines the perception assessment of a phenomenon or object [51,52]. This method is often used in research related to the perception of various elements of human surroundings, e.g., the acoustic environment [53], the color of lighting in gardens [54], the quality of equipment in the public space [55], the attractiveness of the landscape [56], the thermal comfort [57], the visual contamination of the streets [58], safety in terms of pedestrian traffic in the cities [59] or the quality of green areas [60].

**Figure 2:**
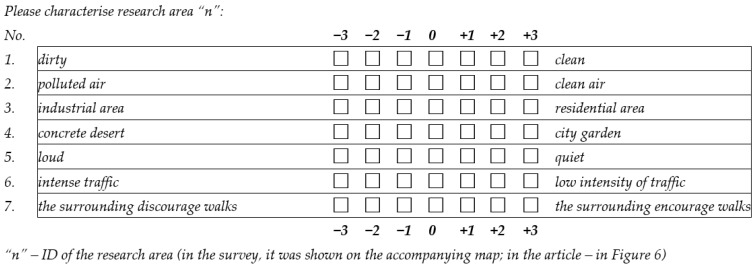
Semantic differential used to evaluate the research areas.

The use of the semantic differential method required answering questions which are presented in the form of a table. Each line of such a table contains a pair of opposite descriptions (e.g., “dirty—clean”) placed on a bipolar scale. The role of the person assessing a phenomenon is to indicate their own perception of the rated phenomenon or object on this scale. To enable the fulfillment of the task, in the case of our study, the respondents were given a seven-degree scale (from −3 to +3), which provided them with the ability to choose an answer unambiguously. Importantly, it also enabled the selection of a neutral middle value (0). The semantic differential used to rate 32 residential neighborhoods is presented in Figure 2.

Although the perception of air quality is usually associated with the assessment of visibility [61] and odor [62], the presented semantic differential does not include pairs of opposite descriptions related to those two most obvious features of the air. There was concern that their assessment may be difficult for a person filling in semantic differentials for the specific areas of a single city. On the other hand, the table lists the pairs of more tangible and more easily assessed features related to the main determinants of air pollution indicated in world research: road transport (Figure 2, line 6), industry (Figure 2, line 3) and heating [63,64,65], which often results in excess noise (Figure 2, line 5) and dust, causing contamination of the surroundings (Figure 2, line 1). Attention was also paid to the fact that the presence of urban greenery can have a positive impact on air quality (Figure 2, line 4) [66]. Moreover, the semantic differential included a question about walkability (Figure 2, line 7). It was assumed that areas better rated in terms of air quality (or in terms of the factors affecting air quality) are definitely more encouraging for traveling on foot. Obviously, there was also a direct question about the general impression related to air quality (Figure 2, line 2). However, one should be aware that the respondents assessing this feature could have been biased by their remaining answers.

The simplest form of analyzing the data collected in the semantic differential is to draw a graphical profile, the vertices of which constitute numerical values which were obtained for the individual pairs of the opposing features [51,52]. In the case of an analysis of data acquired from a group of people, it is best to determine the average values resulting from the answers given by the respondents in each line of the table in which the question is written. When comparing several semantic profiles (in our research there are 32 of them—one for each research area), in addition to the average values, it is also recommended to determine their standard deviations.

In addition to the semantic differentials, the survey form also included control questions and a data sheet. For control purposes, it was requested to indicate which research area fulfills the specific criterion the best and the worst. There were eight of these criteria: (1) the cleanness of surroundings, understood primarily as the absence of dirt and dust; (2) the cleanness of air; (3) the diverse number of spatial functions necessary for proper functioning; (4) the development and surface area of urban greenery; (5) acoustic comfort; (6) friendly intensity of road traffic; (7) walkability; (8) encouragement to live in the area.

#### 2.2.3. Modeling the Level of NO_2_ Generated by Road Transport

Verification of the results obtained in the survey related to the appropriateness of the choice of neighborhoods with bad and good air quality was performed with the use of advanced GIS technologies and the ADMS-Roads computer software for modeling the level of air pollution. Taking into account the research results [41] and the prevalence of road transport as the primary source of gaseous pollutants in city [47], the verification was performed for pollution with NO_2_ generated by the road traffic.

This began by determining the annual average of daily traffic intensities in the road transport network (Annual Average Daily Traffic—AADT volumes in the road transport) along the segments of roads belonging to the two specified residential neighborhoods. AADT is the number of motor vehicles traveling in a given segment of the road during 24 consecutive hours, averaged for a period of one year. The method of its calculation depends on the type of the measurement segment (the P type segment—basic segments where direct measurements of traffic are performed on a full-time basis, and the T type segment—segments of the road where no direct measurements are performed, and the data may be acquired indirectly, by generating them from specialized software) [67]. For the two indicated segments, the value of AADT is to be calculated according to the formula:

(1) $$\mathrm{AADT}=\frac{M_{R}\cdot N_{1}+0.85M_{R}\cdot N_{2}+M_{N}\cdot N_{3}}{N}+R_{N},$$

wherein: *M_R_*—average daily traffic on working days (Monday to Friday, 6 A.M.–10 P.M.), 0.85*M_R_*—average daily traffic on Saturdays and pre-holiday days (6 A.M.–10 P.M.), *M_N_*—average daily traffic on Sundays and holidays, *R_N_*—average night traffic (10 P.M.–6 A.M.), *N*_1_—the number of working days in the year, *N*_2_—the number of Saturdays and days preceding holidays in the year, *N*_3_—the number of Sundays and holidays in the year, *N*—the number of all days in the year. The research used the results of direct traffic measurements made available by the City Road Administration in Kielce. The traffic was measured using the manual method in 42 measurement points located at road junctions and in the internode segments of the most unfriendly roads in Kielce. They were mostly national and provincial roads. A total of 22 streets were examined. The time of performing the measurements corresponded to the time of carrying out surveys among the inhabitants.

The results of traffic measurements were compiled in a GIS database. The geometry of the road network was linked with the descriptive data. To calculate the level of NO_2_ using the ADMS-Roads software, it is necessary to provide a set of information, related not only to the intensity of road traffic or the percentage of heavy vehicles passing through a given segment of the road, but also to characterizing the basic parameters of the analyzed roads (width of the road corridor, speed limit) and to identifying the buildings, located in the frontage. The necessary information was acquired from field research and from spatial data in open resources [68,69,70]. The calculated width of the road corridor constituted a sum of the widths of the road elements and the area necessary for the placement of devices associated with it [71]. The height and the type of the buildings directly adjacent to the road are especially important for the propagation of air pollution. The higher the buildings, with more compact building design, the weaker the aeration of the area, with heavier accumulation of pollutants. The adopted model determined the height of buildings located in the frontage, and assigned them to the respective height groups [72]. According to Section 8 of the resolution, the height of a building is measured from the ground level near the lowest entrance into the building or its part, located on the first aboveground story of the building, to the top surface of the highest roof, including the thickness of the heat insulation and the layer which shields it, not taking into account the motor rooms of lifts and other technical rooms extending above this plane, or the highest point of a flat roof or a covering structure of a building located directly above the rooms designated for accommodating people. The following height groups were distinguished in the model:

- Low buildings (N)—up to and including 12 m above ground level, or residential with a height of up to and including four aboveground stories.
- Medium height buildings (N)—from 12 up to and including 25 m above ground level, or residential with a height of from 4 up to and including 9 aboveground stories.
- High buildings (N)—from 25 up to and including 55 m above ground level, or residential with a height of from 9 up to and including 18 aboveground stories.
- High-rise buildings (WW)—more than 55 m above ground level.

Moreover, the type of building design was determined in accordance with the following classification:

- Low buildings (N)—up to and including 12 m above ground level, or residential with a height of up to and including four aboveground stories.
- Buildings in compact design (Z), meaning such where the buildings fill the entire front of the land lot, leaving no gaps, which prevents or considerably limits the process of aeration.
- Buildings in dispersed design (R), which allows for gaps between buildings with the width of entire buildings, which improves the process of aeration considerably.

The ADMS-Roads software was used to calculate the level of pollution with nitrogen dioxide. ADMS-Roads, a version of the Atmospheric Dispersion Modeling System (ADMS), is a PC-based model of atmospheric dispersion of pollutants released from road traffic and industrial sources. In the modeling of dispersion of road traffic, emissions allowance is made for initial dispersion in the vehicle wake, traffic-induced turbulence, and the effects of street canyons. Compared to some other models used for air dispersion modeling near roads, a significant feature of the model is that ADMS-Roads applies up-to-date parameterizations of the boundary layer structure based on the Monin-Obukhov length, and the boundary layer height. This allows for a realistic representation of the character of dispersion changing with height. Typical applications of the model include the development of air quality action plans, investigation of air quality management and planning options for road transport sources, assessment of low emission zones (LEZs), source apportionment studies as well as air quality and health impact assessments of proposed developments [73].

Air quality models use mathematical and numerical techniques to simulate the physical and chemical processes that affect air pollutants as they disperse and react in the atmosphere [74]. Based on inputs of meteorological data and source information, such as emission rates and stack height, these models are designed to characterize the primary pollutants that are emitted directly into the atmosphere and, in some cases, the secondary pollutants that are formed as a result of complex chemical reactions within the atmosphere. The most commonly used air quality models include Dispersion Modeling. This model is typically used in the permitting process to estimate the concentration of pollutants at specified ground-level receptors surrounding an emission source. The dispersion model varies depending on the mathematics used to develop the model, but all models require an input of data which may include:

- Meteorological conditions, such as wind speed and direction, the amount of atmospheric turbulence (as characterized by what is called the “stability class”), the ambient air temperature, the height to the bottom of any inversion aloft that may be present, the cloud cover and solar radiation.
- Source term (the concentration or quantity of toxicants in emission or accidental release source terms) and temperature of the material.
- Emissions or release parameters such as source location and height, type of the source (i.e., fire, pool, or vent stack) and the exit velocity, exit temperature and mass flow rate or release rate.
- Terrain elevations at the source location and at the receptor location(s), such as nearby homes, schools, businesses, and hospitals.
- The location, height, and width of any obstructions (such as buildings or other structures) in the path of the emitted gaseous plume, surface roughness, or the use of a more generic parameter: “rural” or “city” terrain.

**Figure 3:**
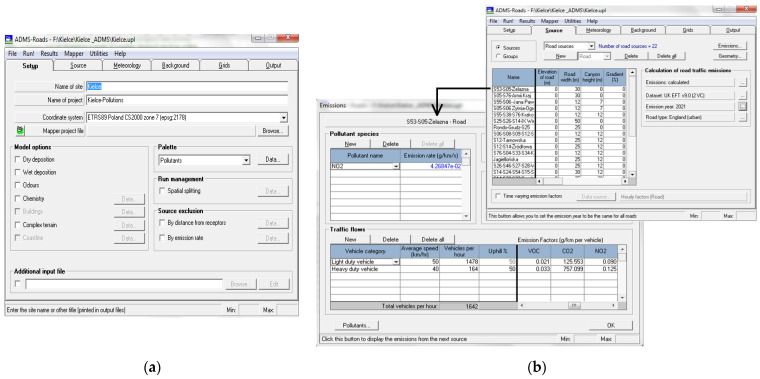
(**a**) Setup of an ADMS-Roads dialogue box without model options; (**b**) ADMS-Roads Emissions dialogue box.

The ADMS Road air dispersion modeling is the study of the transport of air pollutants from a roadway or another linear emitter. Setting up a modeling problem requires the user to input information specifying the release conditions, the meteorological conditions, and the required output. The required minimum input data were used to run the model. In our case, no model options were selected, and the ADMS-Roads calculated concentrations for a pollutant released in flat terrain without taking into consideration the chemical and deposition aspects (Figure 3a).

The ADMS-Roads model calculates emissions from traffic at street level, and it can be applied to approximately calculate various air pollutants, including NO_2_. The software is fully optimized, making it possible to visualize the data on a GIS map, and to make use of road networks connected with the road traffic database, with AADT being an essential parameter for calculating the emission of air pollutants. In ADMS-Roads, the smallest road segment is called a link. The number of links must be at least equal to the number of intersections, and all changes in the road network, such as curves, ought to be represented by separate links. The length of a link has to be sufficient to enable all vehicles to be accounted for during the construction of the model. At the same time, each link contains descriptive attributes regarding the length of the link, the number of vehicles per hour (light and heavy-duty vehicles), the average speed of a vehicle and the characteristics of the link (e.g., elevation, width, canyon height). Examples of dialogue boxes for nitrogen dioxide emissions are presented in Figure 3b.

## 3. Results and Discussion

### 3.1. Analysis of Source Materials Related to the Level of NO_2_ Pollution for Kielce City

#### 3.1.1. Data from the Copernicus Sentinel-5P Satellite

**Figure 4:**
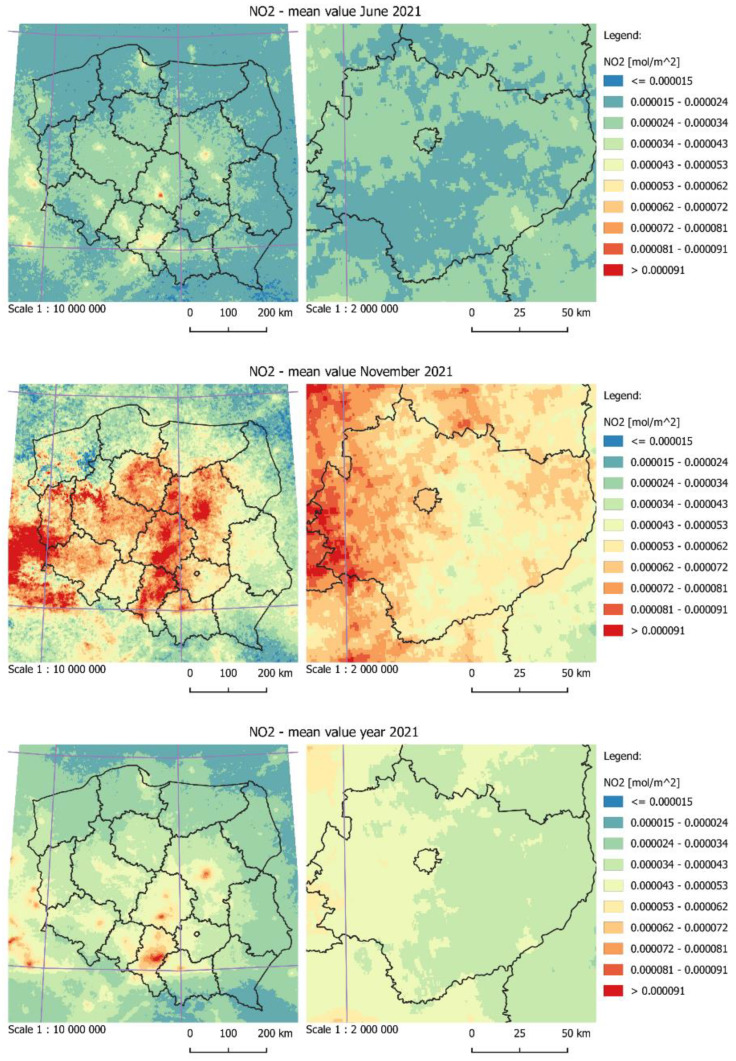
The average value of NO_2_ concentration in June and November, and for the whole year 2021, calculated based on the data from the Copernicus Sentinel-5P satellite.

The evaluation of air quality was performed based on the data recorded by the Copernicus Sentinel-5P satellite. The data were processed and then downloaded by means of the Google Earth Engine platform [46]. Figure 4 presents the data related to a tropospheric vertical column of NO_2_. On the Google Earth Engine platform, the average values of NO_2_ concentration were calculated in June, November and for the whole year 2021. The exported data in the GeoTiff format were subsequently visualized in the QGIS environment. The pictures on the left present the situation for the whole Poland; the pictures on the right address the Świętokrzyskie Voivodeship and Kielce city. In all the figures, there is a clearly visible increase in the concentration of pollutants in the heating month, which results from an increase in the share of point sources (the heating of buildings). In the summer month, the pollution level is three times lower and thought to be generated primarily by linear sources (road transport). In the case of an average yearly value, the highest values of NO_2_ were observed in the area of the Upper Silesian Industrial Region, the Bełchatów Power Plant and large urban agglomerations, such as Warsaw, Krakow, Łódź or Wrocław. Unfortunately, the resolution of data with the abovementioned value of 0.01 degrees does not allow for a detailed analysis of air pollution with respect to the specific neighborhoods of the city or individual streets. Therefore, when using the abovementioned source, it is not possible to internally differentiate the city space and select areas with better or worse air quality. The acquisition of data from the level of the outer space is also hindered by the cloud cover, which effectively obstructs the records of NO_2_. This is especially problematic if the analysis is to focus on the data from the individual days. In this case, the only solution is to determine the average value of pollution in the individual weeks or months.

#### 3.1.2. Official Data

**Figure 5:**
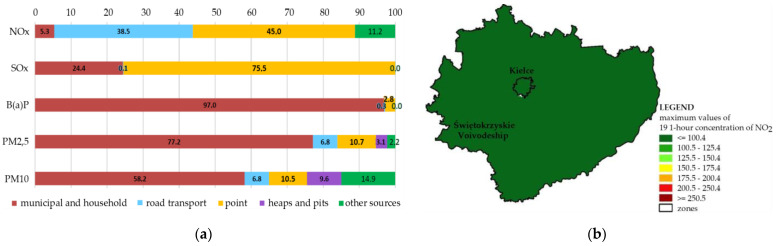
(**a**) Percentages of the emission sources of specific air pollutants in Świętokrzyskie Voivodeship; (**b**) Spatial distribution of maximum values of 19 1-h concentration of NO_2_ in Świętokrzyskie Voivodeship and in Kielce city in 2020.

As indicated in the Report [47], point sources have the highest share in the emission of gaseous pollutants in Świętokrzyskie Voivodeship. In the case of NO_2_ emissions, the share of point sources is 45%, and 38.5% are from road transport (Figure 5a). The data acquired from the monitoring stations indicate that the level of NO_2_ in Kielce was not exceeded, and the city was categorized as Class A. In the city zone, at the NO_2_ measurement station, the maximum 19 1-h concentration was 107 µg/m^3^, and the average annual concentration was 24 µg/m^3^, which constitute 54% and 60% of the valid permitted levels for the protection of human health: 200 µg/m^3^ for the 1-h concentration, and 40 µg/m^3^ for the average annual concentration. The data presented in the report refer to the whole voivodeship, including the city understood as a zone. Therefore, Figure 5b does not show the spatial differentiation of air quality in Kielce city. This makes it impossible to perform a detailed air quality assessment and select areas in the city with better or worse parameters, not only in terms of NO_2_, but also in terms of other types of pollutants.

The “Program for air protection for Świętokrzyskie Voivodeship with a plan of short-term actions” is currently in force in Kielce city [48]. A report prepared for the needs of the abovementioned program indicates a visible trend of air quality improvement in the city in the recent years. Unfortunately, in Kielce there are frequently observed locally considerably exceeded concentrations of pollutants in the air, with respect to the particulate matter and NO_2_. Road transport is the main reason behind the increase in NO_2_ emissions in the city. The Program [48] suggested actions limiting low uncontrolled emission of particulates, among other methods, by the elimination of coal furnaces and by limiting emissions from transport. Since 2017, the city has been handling an inventory of heating systems, which will allow for diagnosing the situation related to the use of energy sources in households. Moreover, information on heating sources in buildings within the city is inventoried and collected. It was estimated that in 6000 single-family residential buildings, 148 multifamily residential buildings and nine public utility buildings belonging to the municipality, there are still heating devices or systems not fulfilling the low emission standards. Unfortunately, no studies are underway in relation to linear sources. The problem of the level of pollutants generated by the road transport has been omitted both in the Report of the Provincial Inspectorate for Environmental Protection [47] and in the Program adopted by the city authorities [48].

Kielce city also offers its inhabitants an air quality monitoring portal which has been created as part of the “Kielce without smog” or “Idea Kielce” projects. This system consists of 21 devices for measuring air quality; however, it does not show the entirety of the situation in the city. The devices used serve only the measurement of the particulate matter, and they do not measure the level of NO_2_ [44,45].

An analysis of the abovementioned documents indicates that the currently performed air quality assessment presented in reports, programs, on web portals, or via an analysis of satellite data, provides no reliable information on the spatial differentiation of the city in terms of pollution with NO_2_. The presented assessment is the result of interpretation of measurements performed at three measurement stations located in the city, one of which constitutes a suburban station and does not reflect the situation in the very center. The results presented in the analyzed sources refer to the entire zone (city), which is in accordance with the current air quality assessment procedure [14]; however, they do not show the internal variability of the distribution of pollutants, which is the result of the type of sources, their characteristics or land morphology and development. Therefore, when analyzing the available source materials, it is impossible to internally classify the city (specific residential neighborhoods) in terms of air quality.

### 3.2. Studying the Perception of Air Quality in Kielce City

**Figure 6:**
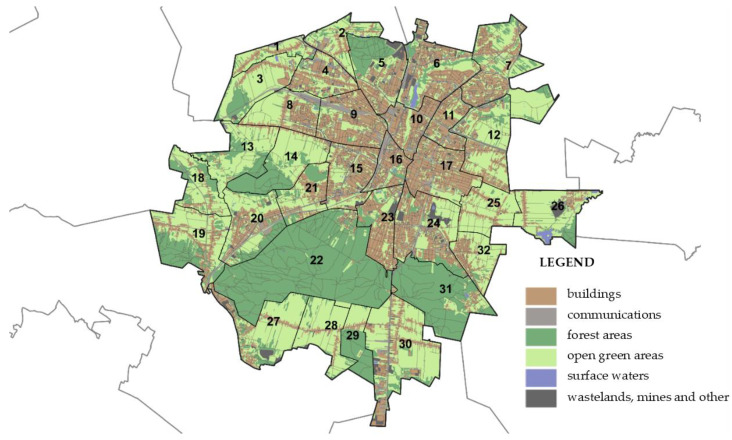
Separate research areas in the city of Kielce.

Spatial differentiation of the city in terms of air quality and the selection of research areas with bad and good air quality were performed using the quantitative semantic differential (SD) method for studying perception assessment. For this purpose, Kielce was divided into 32 units, which were rated in the survey. The shape of the boundaries of these areas complies with the borders of the surveying districts functioning in the city (Figure 6).

The study lasted from 23 June 2021 to 25 November 2021. Therefore, the timeframe of the research took into account the variance of the sources of gaseous pollutants. For point sources (household and municipal—related to the heating of buildings), it took into account the time during the heating season and the time beyond this season. For linear sources (road transport), it took into account the autumn season, meaning the time when road traffic is increased, which is associated with everyday commutes to workplaces or education centers, and the summer season, when there is a considerable reduction of road traffic due to holidays.

The surveys were presented to experts, meaning people closely familiar with Kielce and knowledgeable about air quality and the impact of pollution on the quality of life in the city. Therefore, they were people fulfilling two combined conditions: (1) living in city, or those who live in its close vicinity and travel to it every day in order to fulfill their professional duties; (2) linked with the subjects of air quality by the field in which they are active (physicians, engineers, logisticians, officials, and researchers, who in their work address issues generally associated with air quality). Due to the COVID-19 pandemics, the responses were collected online using the CAWI technique (Computer-Assisted Web Interview). Each respondent was supposed to rate each of the 32 research areas by means of the semantic differential, and respond to the control questions. Answers were received from 108 individuals. This number was deemed sufficient due to the targeted manner of selecting the respondents. Those who decided to fill in and send back the form were more often women (almost 70%), with higher education (over 80%) and living in Kielce (over 80%). Detailed characteristics of the respondents are presented in Table 2.

The respondents rated the air quality in the whole city as good. Such an answer was chosen by almost 60% of the respondents. Interestingly, the results obtained in the survey overlap with the answers given by the users of the NUMBEO portal [75], who so far gave the city a rating of 50.00/100.0, i.e., moderate, in all the possible categories (including air pollution and air quality).

An analysis of the survey results started with reviewing the answers to the control questions (Table 3). The respondents claim that the worst area to live in is area 9. On the other hand, the best one is area 7. The answers given to the remaining questions also suggest that these are the areas which should be analyzed as having the air with the worst and best quality, respectively.

**Figure 7:**
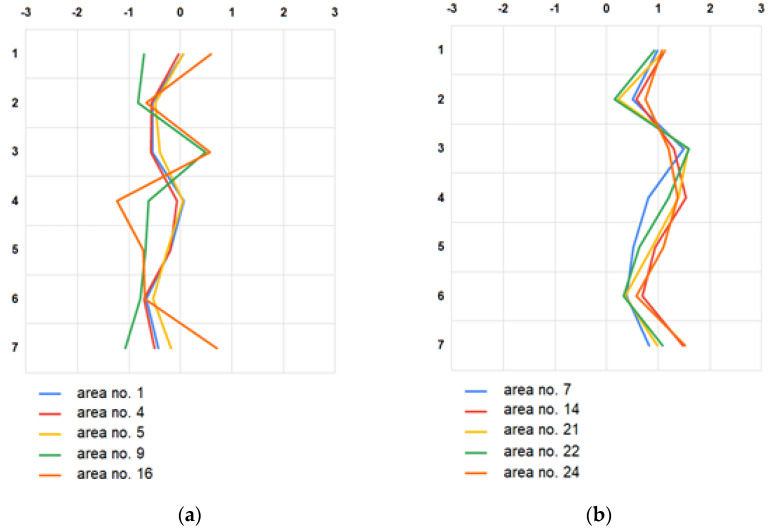
Semantic profiles of the 5 worst (**a**) and best (**b**) rated research areas.

**Figure 8:**
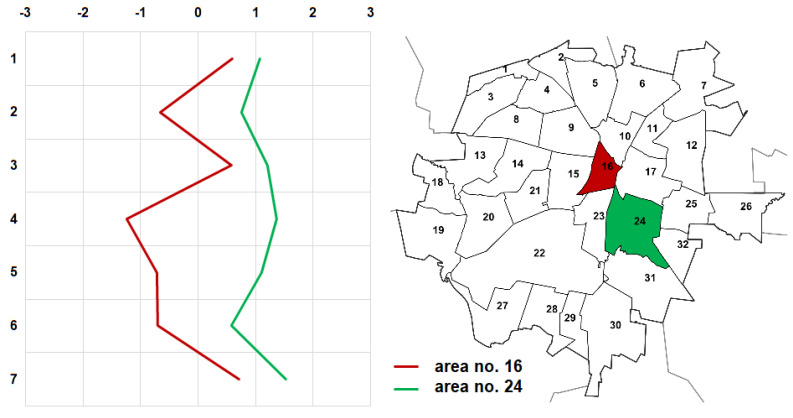
Semantic profiles of areas selected for detailed analyses: area no. 16 (red) rated as one of the worst; area no. 24 (green) rated as one of the best.

The answers given to the semantic differentials were used to calculate basic descriptive statistics (Table 4, Table 5 and Table 6), as well as to draw semantic profiles (Figure 7 and Figure 8). Initially, arithmetic means were determined for the answers given in each question, for each research area (Table 4). To facilitate the analyses of the produced results, a color on a scale from red to green was assigned to each value listed in the table. The most intense red represents the lowest values (min. = −1.23 for area no. 16), with the darkest green for the highest (max. = 1.90 for area no. 11). All red boxes describe areas no. 1, 4 and 5. However, the most intense red was assigned to areas 9 and 16. All green boxes in turn describe areas no. 7, 14 and 24. Areas 21 and 22 also received a high rating.

However, it was found that it would be easier to draw the initial conclusions based on a heat map of the medians from the answers (Table 5). The applied color scheme is identical to the heat map of average answers—the minimum value (−1) is indicated in red, with the maximum (2) in darker green. The highest number of red fields describes areas 9 and 16, with the majority of dark green fields for areas 14 and 24.

The final outcome of the performed surveys is the semantic profiles, in the vertices of which are the average values of the responses given in the semantic differentials. Because of the number of the analyzed areas, Figure 4 presents the semantic profiles of five research areas previously identified as rated the worst (a) and the best (b).

The significance of answers from the performed surveys was verified by means of the standard deviations of the answers given to specific questions, for the individual areas (Table 6).

The resulting values are not satisfactory, but they should not be surprising either. These are perception surveys; therefore, it is not surprising that the standard deviations of responses given in the individual questions are very high, with an average of 1.44 ± 0.13. Therefore, based on the produced results, it would be difficult to unambiguously pinpoint (among the five best and worst) the residential neighborhoods with bad and good air quality. It was thus decided to carry out a reconnaissance, which was supposed to help in this choice. The worst rated areas are located in the north-western part of the city (cf. Figure 2). They include the very center (area no. 16) and other heavily urbanized areas (1, 4, 5 and 9), within which (or in their nearest neighborhoods) there are roads with heavy traffic, railway areas, and/or industrial plants. On the other hand, the best rated areas are located outside the city center. Three of them (7, 21 and 24) are areas comprising mainly residential buildings (both single and multifamily). The remaining ones (14 and 22) are areas of greenery. Based on all the available information, it was concluded that areas 16 and 24 would undergo further analyses (Figure 8). However, it should be highlighted that the results obtained only provide the context regarding the perception of participants to the air quality of Kielce and a basis for further investigation.

Area no. 16 will be analyzed as the one which was rated the second worst in terms of air quality in the city, which was additionally usually described by the respondents as a concrete desert. This choice is also related to the fact that area 16 comprises the very center of the city, in which there is very intense vehicle traffic, and in which there is a compact urban fabric, which potentially obstructs the movement of air and aeration of this part of the city. The high rating for walkability and cleanness in this area are derived from the care for the image of the representative part of the city, rather than actual improvements for people traveling on foot, or the absence of air pollution.

Area 24 will be analyzed as an area with good air quality. Although it received a slightly lower overall rating than area 14, it is the more urbanized one and it definitely contains more roads, which will constitute a basis for further studies. For the purpose of the analysis, it is also important that residential neighborhood no. 24 is located in the direct vicinity of an area rated as one of the worst, meaning residential neighborhood no. 16.

### 3.3. Identification of Areas with Good and Bad Air Quality

The areas designated 16 and 24 were analyzed in detail.

**Figure 9:**
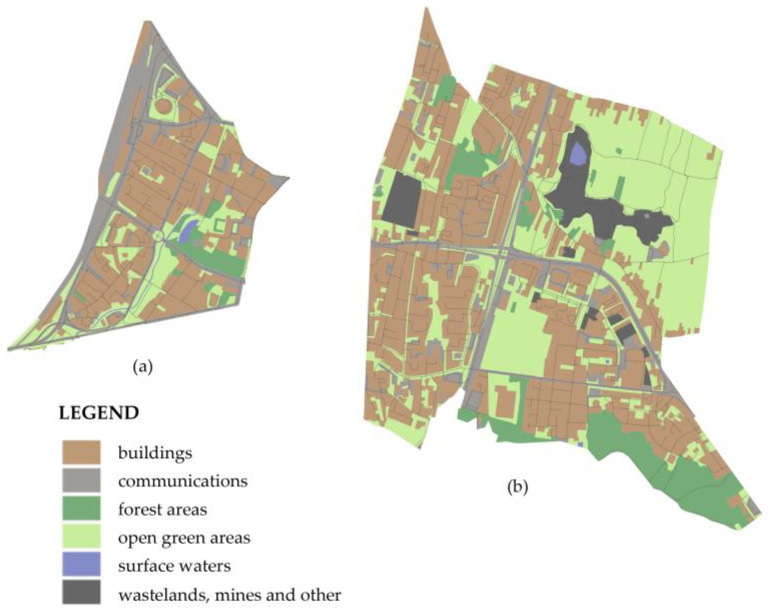
Development of the analyzed research areas: (**a**) area no. 16; (**b**) area no. 24.

Area no. 16 includes the very center of Kielce. It includes the routes of the main transport system, roads of national significance, with an increased share of heavy vehicles (Ogrodowa and Żelazna Streets). The intense traffic is influenced by the presence of two stations: a PKS coach station and a PKP train station, which considerably increase the road traffic (of light and heavy vehicles) and the rail traffic (passenger and cargo rail vehicles) in the analyzed area. Area no. 16 is characterized by compact building design of medium height, constituting a concentration of administrative, cultural, and historical commercial buildings. These are mostly buildings commissioned in the 19th and the 20th centuries, with a moderate technical condition. Most of the administrative objects have undergone thermal upgrading. Within the abovementioned area there are sparse green zones—the Stanisław Staszic City Park and the Szarych Szeregów Scout Square (Figure 9a).

Area no. 24 in turn includes largely green areas, which constitute a major asset of the analyzed space. Two green dominant spatial features can be distinguished in the northern part of the area. The first one of them is the partially woody elevation of the Kadzielnia Range—the Sunny Mountain (commonly known as “Dog Hill”) with the existing remnants of a quarry, on which there is a three-hectare protected area. This area is used by the inhabitants for recreational purposes all year. Another green dominant feature is the area of the Wietrznia Nature Reserve, established in 1999, with a surface area of 17.59 ha. In the reserve there are precious geological outcrops, fossil fauna and a walking trail. To the south, area no. 24 abuts the Telegraf and Bukówka Mountains, next to which a prestigious residential area is located (the Pod Telegrafem neighborhood), with detached single-family buildings of a residential nature. The western part of the area is in turn dominated by multi-family buildings—the Barwinek residential neighborhood. These buildings are from the beginning of the 21st century, and they are characterized by a very good technical condition. It is a trendy neighborhood in the city, with the highest prices for residential real estate in Kielce [76,77]. Moreover that in the north-eastern part of the area there is an undeveloped land, also used by the inhabitants for recreational purposes. Tarnowska and Ściegiennego Streets (segments of national road no. 73) run across the western part of the area. It is a dual carriageway exiting toward Tarnów, with a transit nature, with a high intensity of light and heavy vehicle traffic (Figure 9b).

### 3.4. Modeling of NO_2_ Generated by Road Transport—Verification of Survey Research

**Figure 10:**
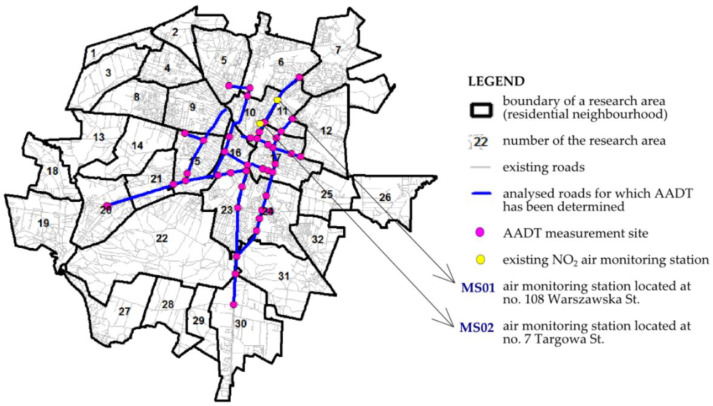
Location of AADT road traffic intensity measurement points, with the position of air monitoring stations against the research areas.

The ADMS-Roads software was used in modeling the level of NO_2_ concentration (µg/m^3^/hour). ADMS-Roads calculated concentrations of pollutants released in flat terrain without taking into consideration the chemical and deposition aspects. Thus, it showed the level of NO_2_ pollution in an approximate manner. The research used the data shared by the City Road Administration in Kielce, which carried out direct measurements of AADT at 42 road junctions, belonging to the 22 most cumbersome roads in the city (Figure 10). The measurements continued from June to November 2021, which corresponded to the time of performing surveys among the inhabitants. The Kielce City Road Administration conducted measurements during the COVID-19 pandemic, which introduced a movement blockade in the city. The introduction of the blockade affected the reduction of motor vehicle traffic. As indicated by studies conducted in other Polish cities during the COVID-19 pandemic, traffic at major intersections dropped by several tens of percent compared to 2019, the time before the pandemic, including in Krakow by 40% [78] and in Wroclaw by almost 30% [79]. As seen in Table 7, most of the investigated roads were of the national and provincial type. The highest intensity of traffic was recorded on Źródłowa St., with the lowest on Jagiellońska St. Out of the 22 roads studied in the city, five were located in neighborhood no. 16 (six measurement sites), and three in neighborhood no. 24 (nine measurement sites). The computational model also took into account the data on the manner of development and the land relief around the roads. The land around the roads was flat, covered with low and medium high buildings in a dispersed arrangement. Compact building design appeared only in the vicinity of Jana Pawła II and Seminaryjna Streets (Table 7).

**Figure 11:**
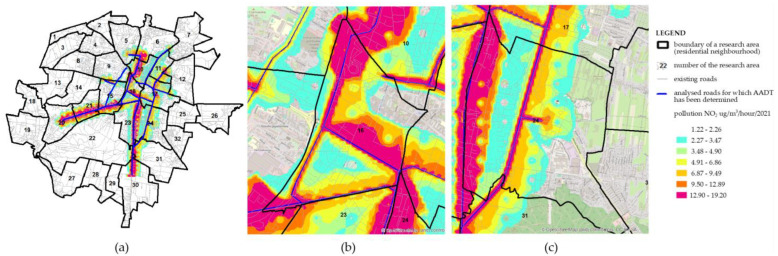
Map of predictions for NO_2_ generated by the roads investigated in Kielce city in 2021; (**a**) situation for the 12 studied research areas; (**b**) situation for area no. 16; (**c**) situation for area no. 24.

As seen from Figure 10, modeling of the NO_2_ concentration level was performed within 12 research areas. This level is the highest near the streets (it amounts to a maximum of 19.20 µg/m^3^/hour), and it drops along with an increase in the distance from the source, reaching a final value within a range of 1.22–2.26 µg/m^3^/hour (Figure 11a). When analyzing the whole situation in the city, in none of the points were there any recorded cases of exceeding the permitted value, which for 1-h concentrations cannot exceed 200 μg/m^3^. Interestingly, the pollution level for research areas no. 16 and no. 24 is satisfactory and comparable. Therefore, the intensity of traffic on the analyzed roads generates a similar level of pollution. The only difference was found in the percentage of areas with a pollution level from the upper interval. Due to the type of building design and the spatial arrangement of the transport system, i.e., a high concentration of roads with heavy intensity of traffic, approx. 40% of area no. 16 is under the impact of NO_2_ with a range of 12.90–19.20 µg/m^3^/hour (Figure 11b). For area no. 24, this percentage is approx. 10. In addition, in area no. 24, the pollutants are concentrated in its western part, outside the residential areas (the “Pod Telegrafem” and “Barwinek” residential neighborhoods) or the green areas of recreation and leisure, improving the spatial values. This may be the evidence of a well-planned area, where the spatial structure guarantees comfort, and the level of air pollution is low (Figure 11c).

**Figure 12:**
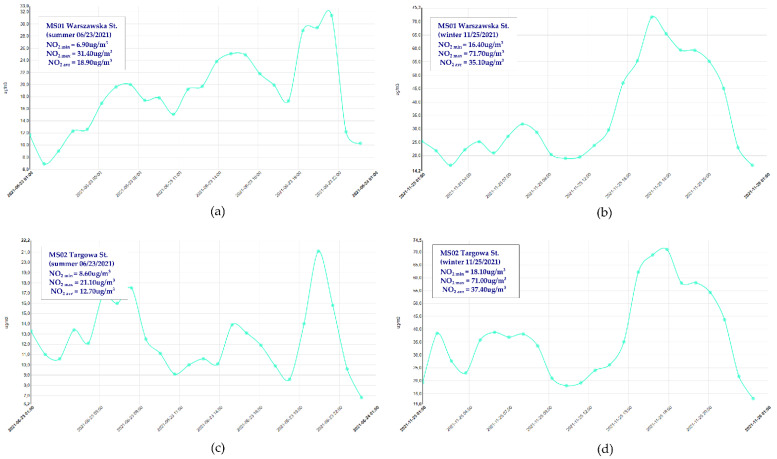
Daily measurement of the NO_2_ level at the air monitoring stations in Kielce: (**a**) station MS01 Warszawska St.—summer season; (**b**) station MS01 Warszawska St.—winter season; (**c**) station MS02 Targowa St.—summer season; (**d**) station MS02 Targowa St.—winter season.

**Figure 13:**
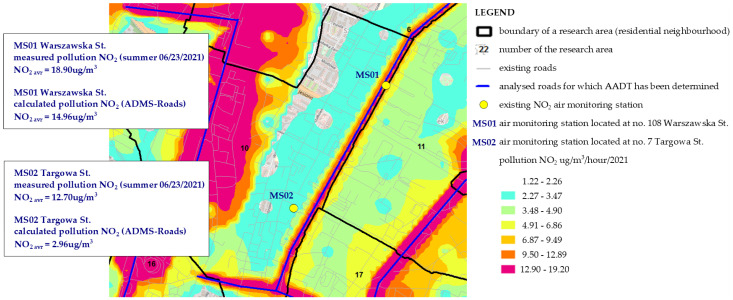
NO_2_ level measured at two air monitoring stations against the calculated level of NO_2_ generated from road transport.

This was followed by the comparison of NO_2_ concentrations measured at two air monitoring stations (placed within the boundaries of area no. 10) (Figure 12) with the data resulting from the prediction model calculated for linear sources (Figure 13). The data were read from two air monitoring stations and for two seasons, which correspond to the dates of beginning and ending the surveys, presenting the variance of the sources of gaseous pollutants [42,47]. The level of NO_2_ pollution shown in Figure 12 is representative of the adopted survey periods. As can be seen, the levels of pollution with NO_2_ for the two stations are similar. The average hourly level of NO_2_ in the summer season (the level read on 23 June 2021) was 18.9 µg/m^3^ for station MS01 and 12.7 µg/m^3^ for station MS02 (Figure 12a,c). This level did not exceed the permitted value. In the heating season (November) the level of the analyzed pollutant doubled, and on 25 November 2021 it amounted to MS01—35.1 µg/m^3^ and MS02—37.4 µg/m^3^, respectively (Figure 12b,d). What is important, is that the permitted level was still not exceeded. This increase is the result of the growing share of point sources in the emission of gaseous pollutants, especially in terms of the presence of high-emission energy sources in households.

Therefore, it can be concluded that measurements in the summer season result largely from the emission generated by linear sources, meaning road transport, which will be the very thing constituting a value for comparing the level measured at the station with the level calculated in the prediction model. As seen from Figure 13, the measured level of NO_2_ is comparable for station MS01, located on Warszawska St. The level measured for MS01 is NO_2_ = 18.9 µg/m^3^; the level calculated in the NO_2_ model = 14.96 µg/m^3^. The measurement at station MS02, 12.7 µg/m^3^, is not comparable to the level obtained in the model, 2.96 µg/m^3^. This may be caused by the fact that direct measurements of AADT, which form a basis for calculating NO_2_, were performed on various days of the week, and the magnitude of AADT in the area of Targowa Street was determined on Friday, when the car traffic is reduced.

## 4. Conclusions

The problem of pollutants originating from road traffic is currently not well examined, and it constitutes a research subject in numerous countries at a local and global level. The investigation of this problem should be a priority, especially in cities classified as a Smart City, which should guarantee a high quality of life of the inhabitants. Based on the results of the analyses and the detailed air quality assessment performed in Kielce city for the selected research areas, a method of spatial diversification of the city has been developed toward the goal of standardization of procedures used to improve environmental monitoring and to expedite the process of acquiring data for increasing the efficiency of actions related to air protection. Based on the research results, the following detailed conclusions can be presented:

- The analysis of available sources does not allow for a detailed evaluation of Kielce in terms of air quality. The spatial (inner) differentiation of the city in that regard requires the application of modern research methods, combining the advanced GIS technologies with the air pollution modeling system.
- The performed modeling of NO_2_ confirmed that the greatest accumulation of pollution exists near a linear source, and this level drops along with an increase in the distance from the road. In Kielce city, there were no recorded cases of exceeding the permitted NO_2_ pollution level during this study. The level of contamination was determined for traffic determined by the City Road Administration in Kielce during the COVID-19 pandemic, which caused significant traffic restrictions. Therefore, a similar study would also need to be performed during the time after the pandemic. Verification of survey results would also need to be carried out for other sources of air pollution, such as point pollution associated with household heating. The above issues will be the subject of future studies.
- When performing a spatial (inner) air quality assessment in the city, local experts primarily analyzed the direction of development of a given area, and they followed well established opinions (e.g., the trend of moving to a specific location in the city). This is confirmed by the results of surveys. As a fragment of the city with the best parameters in terms of air quality, the respondents chose area no. 24, characterized by the highest share of green areas and being filled with villas. A portion of this area is used for the purposes of recreation and leisure. This is an area perceived by the inhabitants as prestigious, considered to be comfortable to live in and characterized by the highest prices for residential real estate. The area chosen by the respondents as the one with the worst parameters is located in the very center of the city, where there are very few green areas (e.g., lawns and squares), and the buildings are dense and often require thermal upgrading. Therefore, it can be concluded that the outcome of the surveys only confirms the perception of the city space, and it does not result from the analysis of the pollution sources.
- According to the local experts, point sources are the main cause of air pollution (household and municipal—related to the heating of buildings), and the heating season (in Poland from October until the end of April) is a time with worse air quality in the city. This can be seen in the results of the surveys, in which area no. 16 has been considered the one with the worst parameters in terms of air quality. The majority of this area is covered with buildings from the 19th century as well as from the 1960s and 1980s in compact design, with a moderate technical condition, which are heated with high-emission fuel.
- The road transport is not perceived by local experts as an emitter of air pollution in the city. This can be seen in the results of the surveys. The first issue is related to the choice of an area with the best parameters in terms of air quality. The chosen area is no. 24, which is filled with detached houses in a very good technical condition, where the main heat source is gas supplied by the city network. In their opinions, the respondents did not take into account the fact that area no. 24 in its central and western parts has two roads of a state-level significance running through it, which are the main north-south routes, and which are the places of intense transit traffic. The second issue is related to the fact that on the streets forming the transport system of areas with the best and the worst parameters, there is similar intensity of road traffic. The streets of the indicated areas are considered to be under the heaviest load of road traffic, and the city authorities monitor them in a continuous manner. Therefore, these streets generate a similar level of pollution. Local experts did not take this fact into account in their responses, and rated these two spaces differently. This is also confirmed by the verification performed using the ADMS-Roads software. The calculated concentrations of NO_2_ for the two selected areas are similar. Therefore, the air quality in these areas is similar.
- The respondents in the survey were local experts, meaning people closely familiar with Kielce and with good knowledge about air quality and the impact of pollution on the quality of life in the city. Unfortunately, the research results confirm that despite being familiar with the problem of environmental pollution, these people are unable to properly identify the pollution sources, and through them pinpoint the areas of the city with good and bad air quality. When analyzing the above, it can be concluded that since these people cannot assess the space correctly, then the remaining community (people who do not deal with the problems of the environment and health as part of their profession or hobby) would likely not be able to properly assess the space in terms of air quality either. This indicates that despite numerous advertising campaigns organized by the city authorities, there is still very low awareness about the problem of air quality in society, especially in the context of identifying the sources of pollution. This conclusion requires the performance of further surveys with a larger research sample, where the respondents would be the average users/inhabitants.

The presented method approaches the problem of air pollution in the city in an innovative manner, since it takes into account not only the objective (measurement- and modeling-based) dimension, but also the subjective dimension in the form of the inhabitants’ opinions on air quality in their city. According to the authors, complicated dependencies between emissions and effects require a systemic approach, involving modeling and computer aided decisions on rationally reducing the pollutant emissions, both at local, national, international, and even global levels, which is also confirmed by the research performed by Badyda’s team [38].

## Figures and Tables

**Table 1:** Specification of air monitoring stations in Kielce city.

No.	Location	Start Date of the Measurements	Measured Types of Pollutants
MS01	no. 108 Warszawska St.	1 January 2021	nitrogen dioxide, benzene, carbon monoxide, PM10
MS02	no. 3 Targowa St.	1 July 2018	nitrogen dioxide, nitrogen oxides, sulfur dioxide, ozone, benzene, PM10, PM2.5, the presence of metals in PM10
MS03	no. 7 Jurajska St.	1 January 2021	PM10, benzo(a)pyrene in PM10

**Table 2:** Characteristics of respondents.

Feature	Characteristic	Number	Value [%]
gender	female	75	69.44
	male	33	30.56
place of residence	Kielce	89	82.41
	the nearest neighborhood of Kielce	19	17.59
age (in years)	under 30	34	31.48
	31–50	38	35.19
	over 50 years old	36	33.33
education	high school education	18	16.67
	higher (university)	90	83.33
air quality in Kielce	very good	7	6.48
	good	62	57.41
	bad	32	29.63
	I can’t judge	7	6.48

**Table 3:** Number of times when specific areas were chosen in the control questions.

No.	Span	Area																															
		1	2	3	4	5	6	7	8	9	10	11	12	13	14	15	16	17	18	19	20	21	22	23	24	25	26	27	28	29	30	31	32
1	max	1	2	0	0	0	1	19	6	0	1	10	0	0	8	0	15	1	2	1	1	2	11	11	8	1	1	0	0	0	2	1	3
	min	2	4	0	2	3	2	0	2	40	1	5	3	0	0	4	18	0	2	2	6	1	0	5	0	1	2	0	1	1	0	1	0
2	max	0	0	3	0	0	0	26	0	1	1	4	4	3	13	0	3	0	2	5	0	1	6	7	13	0	1	0	2	0	1	11	1
	min	0	1	2	4	5	4	1	4	13	2	1	0	0	0	1	37	1	1	1	8	0	4	4	1	1	0	5	1	1	5	0	0
3	max	0	1	0	0	0	0	3	21	1	3	12	0	0	1	2	44	2	0	0	0	0	1	11	2	0	1	0	0	1	1	1	0
	min	1	10	4	2	1	4	8	3	5	0	3	2	1	1	0	6	0	4	3	4	0	7	2	0	2	8	5	6	3	7	5	1
4	max	0	1	2	0	0	1	9	2	0	3	5	0	1	22	0	1	0	0	3	1	3	18	12	12	0	0	0	0	4	1	7	0
	min	0	1	0	2	0	2	3	6	6	7	4	2	0	0	1	66	1	1	1	0	0	0	1	1	1	1	0	0	0	1	0	0
5	max	1	2	1	1	1	0	16	1	1	1	2	2	1	19	0	0	0	2	2	2	4	13	5	9	3	4	1	1	3	2	5	3
	min	0	1	1	5	3	1	3	3	16	7	2	0	0	0	0	52	4	0	0	1	0	1	4	0	0	0	2	0	0	1	1	0
6	max	0	6	5	1	0	2	5	1	0	0	1	1	4	15	0	8	0	2	1	2	3	10	2	8	9	5	1	4	4	1	3	4
	min	0	1	0	3	3	6	1	3	15	5	6	0	1	0	0	45	4	0	0	1	0	0	3	1	0	2	0	0	0	6	2	0
7	max	0	1	1	1	0	0	5	7	0	3	9	1	0	8	0	29	0	2	2	0	3	14	11	5	1	1	0	0	1	1	1	1
	min	0	4	2	3	1	3	3	3	27	5	4	2	1	2	1	3	4	4	3	6	0	3	2	2	1	6	1	3	2	7	0	0
8	max	0	1	0	1	1	1	25	6	1	3	9	2	1	10	0	8	2	1	4	0	1	10	8	5	3	0	0	0	0	0	3	2
	min	3	2	1	4	1	1	1	6	57	1	0	0	2	0	2	14	1	0	0	2	0	0	1	1	0	1	1	2	1	3	0	0

**Table 4:** Heat map of the arithmetic means from the answers given in the survey.

No.	Area																															
	1	2	3	4	5	6	7	8	9	10	11	12	13	14	15	16	17	18	19	20	21	22	23	24	25	26	27	28	29	30	31	32
1	0.06	0.16	0.36	−0.03	0.06	0.58	0.98	0.80	−0.71	0.53	0.90	0.36	0.58	1.13	0.12	0.60	0.33	0.20	0.54	0.31	1.11	0.93	0.74	1.07	0.64	0.47	0.11	0.38	0.50	0.22	0.84	0.79
2	−0.54	0.00	0.09	−0.56	−0.48	−0.07	0.50	0.14	−0.82	−0.16	0.31	−0.04	0.07	0.58	−0.32	−0.66	0.16	0.02	−0.03	−0.43	0.23	0.16	−0.04	0.76	0.22	−0.01	−0.19	−0.08	0.22	−0.26	0.40	0.08
3	−0.55	0.10	0.58	−0.57	−0.39	1.34	1.49	1.53	0.49	1.28	1.90	0.11	0.55	1.31	1.28	0.58	0.76	0.53	0.42	−0.37	1.58	1.59	1.42	1.21	1.06	0.43	0.25	0.52	0.74	−0.09	1.11	1.17
4	0.07	0.43	0.60	−0.07	0.06	0.62	0.82	0.25	−0.61	0.57	0.64	0.48	0.79	1.54	0.55	−1.23	0.30	0.39	1.12	0.71	1.39	1.21	0.88	1.37	0.66	0.53	0.52	0.68	0.90	0.43	1.35	0.84
5	−0.19	0.33	0.40	−0.19	−0.26	0.22	0.51	0.12	−0.68	0.00	0.06	0.17	0.52	0.94	0.13	−0.71	0.14	0.28	0.42	0.10	0.88	0.64	0.18	1.11	0.59	0.58	0.21	0.22	0.42	−0.21	0.64	0.66
6	−0.66	0.04	0.18	−0.71	−0.53	−0.11	0.39	−0.08	−0.77	−0.08	0.01	−0.30	0.17	0.70	−0.24	−0.70	−0.19	0.15	−0.14	−0.15	0.37	0.33	−0.02	0.58	0.43	0.09	−0.34	−0.06	−0.04	−0.41	0.19	0.28
7	−0.42	0.22	0.45	−0.50	−0.18	0.44	0.83	0.42	−1.07	0.17	0.53	0.07	0.44	1.49	0.05	0.72	0.36	0.10	0.68	0.10	0.98	1.08	0.64	1.53	0.43	0.02	−0.01	0.20	0.62	−0.10	1.16	0.46

**Table 5:** Heat map of the medians from the answers given in the survey.

No.	Area																															
	1	2	3	4	5	6	7	8	9	10	11	12	13	14	15	16	17	18	19	20	21	22	23	24	25	26	27	28	29	30	31	32
1	0	0	0	0	0	1	1	1	−1	1	1	0	0	1	0	1	0	0	0	0	1	1	1	1	1	0	0	0	0	0	1	1
2	0	0	0	0	0	0	1	0	−1	0	0	0	0	1	0	−1	0	0	0	0	0	0	0	1	0	0	0	0	0	0	0	0
3	−1	0	0	−1	0	2	2	2	0	2	2	0	0	2	1	0	1	0	0	−1	2	2	2	1	1	0	0	0	0	0	1	2
4	0	0	0	0	0	1	1	0	0	1	1	0	1	2	1	−1	0	0	1	1	1	1	1	2	1	1	0	1	1	0	2	1
5	0	0	0	0	0	0	0	0	−1	0	0	0	0	1	0	−1	0	0	0	0	1	1	0	1	0	0	0	0	0	0	1	1
6	−1	0	0	−1	0	0	0	0	−1	0	0	0	0	1	0	−1	0	0	0	0	0	0	0	0	1	0	0	0	0	0	0	0
7	0	0	0	0	0	1	1	0	−1	0	1	0	0	2	0	1	0	0	1	0	1	1	1	2	0	0	0	0	1	0	1	0

**Table 6:** Standard deviations of the responses given in the survey.

No.	Area																															
	1	2	3	4	5	6	7	8	9	10	11	12	13	14	15	16	17	18	19	20	21	22	23	24	25	26	27	28	29	30	31	32
1	1.34	1.28	1.17	1.34	1.35	1.30	1.45	1.42	1.63	1.43	1.37	1.42	1.20	1.43	1.44	1.39	1.32	1.44	1.31	1.21	1.28	1.27	1.31	1.38	1.35	1.30	1.40	1.30	1.24	1.39	1.15	1.26
2	1.37	1.43	1.48	1.40	1.28	1.50	1.67	1.58	1.40	1.36	1.52	1.38	1.41	1.55	1.33	1.58	1.25	1.41	1.54	1.56	1.59	1.75	1.68	1.53	1.55	1.42	1.40	1.33	1.43	1.45	1.56	1.51
3	1.50	1.50	1.36	1.57	1.51	1.60	1.49	1.57	1.57	1.54	1.34	1.64	1.26	1.40	1.34	1.60	1.47	1.20	1.56	1.74	1.28	1.47	1.49	1.54	1.43	1.45	1.58	1.49	1.35	1.77	1.49	1.56
4	1.24	1.36	1.40	1.31	1.30	1.29	1.59	1.50	1.42	1.43	1.44	1.21	1.32	1.49	1.22	1.48	1.36	1.31	1.27	1.29	1.27	1.41	1.31	1.43	1.38	1.32	1.26	1.27	1.46	1.42	1.33	1.28
5	1.37	1.34	1.24	1.34	1.47	1.53	1.68	1.68	1.42	1.52	1.52	1.49	1.19	1.58	1.28	1.69	1.39	1.34	1.45	1.48	1.34	1.59	1.52	1.54	1.45	1.36	1.38	1.35	1.51	1.46	1.32	1.44
6	1.32	1.46	1.47	1.40	1.43	1.59	1.66	1.74	1.58	1.55	1.57	1.31	1.29	1.53	1.49	1.88	1.38	1.37	1.46	1.58	1.52	1.58	1.51	1.55	1.35	1.57	1.35	1.39	1.48	1.59	1.51	1.32
7	1.45	1.44	1.35	1.43	1.38	1.43	1.49	1.66	1.53	1.48	1.54	1.48	1.30	1.43	1.39	1.61	1.38	1.26	1.46	1.57	1.52	1.64	1.61	1.55	1.47	1.45	1.38	1.33	1.41	1.43	1.29	1.50

**Table 7:** Characteristics of the analyzed roads along with the characteristics of building design in the research areas.

Name of the Street	Road Category ^1^	Road Width [m]	Speed [km/h]	AADT [n/h]	Heavy Vehicles [n/h]	Building Height [m]	Height Group of Buildings ^2^	Type of Building Design ^3^
Streets belonging to area no. 16								
Żelazna	W	30	50	1642	164	none	none	none
Armii Krajowej	W	30	50	656	65	none	none	none
Jana Pawła II	P	12	50	1540	154	7	N	Z
Żytnia	G	12	50	1140	114	7	N	R
Krakowska	P	12	50	433	43	12	N	R
Krakowska	W	12	50	1739	261	12	N	R
Streets belonging to area no. 24								
Tarnowska	K	25	50	1119	111	12	N	R
Popiełuszki	K	25	70	1214	182	12	N	R
Ściegiennego	P	12	40	1562	156	7	N	R
Streets belonging to the remaining areas								
IX Wieków Kielc	G	50	50	799	80	12	N	R
Seminaryjna	G	12	40	484	48	12	N	Z
Źródłowa	K	25	50	2019	302	12	N	R
Jagiellońska	P	25	50	342	34	12	N	R
Warszawska	P	25	50	662	65	12	N	R
Solidarności	K	30	50	1745	261	12	N	R
Sandomierska	G	25	50	502	50	20	SW	R
Zagnańska	K	30	50	1679	168	7	N	R
Łódzka	K	25	50	1785	178	7	N	R
Grunwaldzka	W	25	50	1600	160	12	N	R
W. Pileckiego	W	30	50	917	46	12	N	R

^1^ Road category: k—national, w—provincial, p—county, g—municipal. ^2^ Height group of the buildings: none—no buildings in the nearest vicinity; N—low buildings, SW—medium high buildings. ^3^ Type of building design: Z—compact building design; R—dispersed building design.

## Data Availability

Not applicable.
